# Supplementary material for: Clinicopathological correlation of kidney disease in HIV infection pre- and post-ART rollout
Source: PLoS One. 2022 May 31;17(5):e0269260. doi: 10.1371/journal.pone.0269260 (PMC9154109; doi:10.1371/journal.pone.0269260)
Supplement: Attachment — Submitted filename: Response to Reviewers.pdf [file pone.0269260.s001.pdf]

Division of Nephrology

Department of Internal Medicine

University of Witwatersrand

Johannesburg, South Africa

24/04/2022

PLOS ONE

To the Academic Editor and Reviewers

Re: Response letter for re-submission of the manuscript to PLOS One: Clinicopathological correlation of kidney disease in HIV infection pre- and post-ART rollout

Thank you for your review and pertinent comments regarding our manuscript. We have addressed each requirement and provided a point-by-point response to the issues raised during the review process.

Journal Requirements

1. Style requirements

The manuscript has been formatted to meet the PLOS ONE style requirements (including figures and tables). All changed are tracked in the 'Revised Manuscript with Track Changes'.

2. Ethics statement

The Methods section of the manuscript has been updated to include additional details regarding participant consent.

Ethics clearance was obtained in writing from the Human Research Ethics Committee (Medical) of University of the Witwatersrand, Johannesburg, South Africa; clearance certificate numbers M1511104, M121184, M120874. This is the Ethics Committee affiliated with the academic institution at which the study was conducted.

This approval permitted a record review of all HIV-positive patients who underwent a kidney biopsy at Charlotte Maxeke Johannesburg Academic Hospital and Chris Hani Baragwanath Academic Hospital within the defined study period. Informed consent was waived due to the retrospective study design. All data was anonymized prior to statistical analysis.

3. Data Availability statement

The final minimized data set underlying this study is available via WIReDSpace, the data repository affiliated with the University of Witwatersrand. https://doi.org/10.54223/uniwitwatersrand-10539-32854

Response to Reviewer comments

Reviewer 1

1. It's not clear that all patients who were biopsied after ART rollout were receiving and taking ART. Plasma viral loads would address this issue. If this is not available this should be acknowledged as a limitation.
   A sentence noting HIV VL availability and results has been added. HIV VL was available for 247 patients (9 of these were from the pre-ART rollout group). Seventy-three patients had HIV VL lower than detectable limits (Lines 132 - 135, 'Revised with Track Changes' manuscript). ART use data was only available for 196 patients (of the 229 patients in the subgroup), 81 (41.3%) of these patients were on ART, of which 41 patients were virally suppressed (data added to table 4, line 205 'Revised with Track Changes' manuscript). This is now noted in the limitations of the study.

2. Whether biopsies were performed even with small echogenic kidneys. Prompted by the fact that 32% had eGFR <15ml/min/1.73m².
   Presence of small kidneys is an exclusion criterion for kidney biopsies at both centers where the study was conducted. Biopsy of small kidneys is associated with increased risk of complications and thus was avoided.

3. Biopsy complications and useful to know how the kidneys were visualized and size of biopsy needles.
   At both centers kidney biopsies are performed under ultrasound guidance using size *16G* automated biopsy needles. Overall complication rates are low but exact data is not available for this cohort.

4. It's not clear whether Table 3 provides a single diagnosis for each patient or whether the patient could have had two diagnoses.
   The Methods section outlines that 'biopsies with multiple diagnoses were assigned its major clinical-pathological diagnosis for the purposes of analysis' (Line 104, 'Revised with Track Changes' manuscript).

5. All biopsies were read as HIVAN or FSGS (NOS). This would mean there were no cases of peri-hilar, tip or cellular variant FSGS or alternatively the term FSGS (NOS) may be used to mean all non-collapsing variants.
   Histological diagnoses were tabulated using the 2018 Kidney Disease Improving Global Outcomes (KDIGO) Controversies Conference guidelines. These guidelines refer to the term 'FSGS (NOS) in the setting of HIV' to describe all non-collapsing forms of FSGS. This has been clarified in the Methods section (Line 101, 'Revised with Track Changes' manuscript).

6. Patients were grouped into pre and post ART rollout. Viral loads were similar in the two groups. It would be useful to know the fraction with full viral suppression for each diagnosis in the post-ART era.
   Missing data limits the interpretation on viral loads in the post-ART rollout group. In the entire cohort HIV VL was available for 247 patients (only 9 of these were from the pre-ART rollout group); 73 (29.6%) had viral loads lower than laboratory detectable limit (40copies/ml). The patients with HIV VL lower than the detectable limit were excluded from the calculation of median viral loads shown in Table 1. A paragraph (Lines 183 - 188 in the

‘Revised and Track Changes’ manuscript) and Fig 3 (Line 195 in the ‘Revised and Track Changes’ manuscript) have been added to show the percentage of patients achieving viral suppression among glomerular dominant lesions. In the post-ART rollout subgroup of 229 patients, 196 patients had data available regarding ART use, 81 (41.3%) were receiving ART at the time of biopsy and 41 (50.6%) of those patients were virally suppressed.

7. Minor comments
    a. Replace HIV-infected with HIV-positive
       Changes made to manuscript as suggested
    b. Use sex (biology) instead of gender (social role)
       Changes made to manuscript as suggested
    c. Delete ‘retrospective’ in the sentence on page 9
       Retrospective deleted
    d. Some confusion between no. of cases and fraction of the study group
       The word ‘number’ changed to ‘fraction’ to assist with clarity (Line 170 of ‘Revised with Track Changes’ manuscript).

Reviewer 2

Clinical data needs some clarification because they are not clearly defined in Methods section and only as results

1. Clinical data (renal function, CD4 count and HIV VL) – when were they collected? What is the actual sample size for each of these parameters? Missing data?
   The Methods section details the following clinical parameters were collected at time of kidney biopsy for the entire cohort: serum CD4 count, HIV viral load, serum creatinine, eGFR, urine protein creatinine ratio, indication for biopsy and renal histological pattern. The sample size of available data for each parameter has now been noted in the text of Results section (Lines 127 – 135 of ‘Revised with Track Changes’ manuscript). In the subgroup of 229 patients the following additional parameters were collected at time of biopsy: serum hemoglobin, serum albumin and ART use. The sample size of available data for each parameter has now been noted in Table 4 of Results section (Line 205 of ‘Revised with Track Changes’ manuscript). In the subgroup serial creatinine and eGFR measurements were collected (retrospectively) at the following time periods after the biopsy: 3 months, 6 months and then yearly for years one to five.

2. Despite the introduction of ART in 2004, how many patients were actually taking the drugs? Authors mention that by 2010, only 55% of the patients were taking drugs. They also mention on line 161, pg. 9, that only 81 pts were taking ART (13% of post ART sample). If this is the case, very few patients were on ART treatment which certainly have little impact on the outcome of the majority of patients included in the study.
   Data relating to ART use was available in the subgroup of 229 patients (all post-ART rollout). In this subgroup, 196 (85.6%) patients had data available regarding ART use, 81 (41.3%) were receiving ART at the time of biopsy. The missing data limits our ability to draw conclusions of ART use in this cohort. Median CD4 count increased year-on-year after rollout; the percentage of patients with CD4 count below 200 x 106 cells/L decreased from 60.5% before ART rollout to 44.2% after rollout ($p = 0.051$). This may be an indication that there were more than the 81 documented patients on ART in this subgroup. This limitation is now noted in the discussion.

3. Renal survival – it's not clear the duration of follow up. The following needs clarification: how many patients were analysed for renal survival (sample size, pre or post ART rollout, treated or not treated). How they analysed renal survival in 5 years.
   In the post-ART rollout group serial serum creatinine and eGFR measurements were available in the subgroup of 229 patients. Renal survival, defined by an eGFR above 15mL/min/1.73m^2, censored for patient default with preserved function, was fitted for 229 patients biopsied post-ART rollout (with follow-up data) using the Kaplan Meyer method; histological diagnoses were compared using Log-rank testing (Fig 5, Line 229 of 'Revised with Track Changes' manuscript). The Methods section has been updated to make this clearer. Due to this being a retrospective study there were many patients lost to follow up (median duration of follow up was 3 months after biopsy (IQR 0 – 36 months)).

Thank you for considering our manuscript. We hope to have addressed each requirement appropriately and receive a favorable response to this resubmission.

Yours sincerely

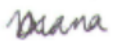

Dr. Nina Diana
